# Development of Glucose Transporter (GLUT) Inhibitors

**DOI:** 10.1002/ejoc.201901353

**Published:** 2019-11-28

**Authors:** Elena S. Reckzeh, Herbert Waldmann

**Affiliations:** ^1^ Department of Chemical Biology Max Planck Institute of Molecular Physiology Otto‐Hahn‐Str. 11 44227 Dortmund Germany; ^2^ Department Chemistry and Chemical Biology TU Dortmund University Otto‐Hahn‐Str. 4a 44227 Dortmund Germany

**Keywords:** Antitumor agents, Cancer, GLUT inhibitors, Natural products, Virtual screening

## Abstract

The discovery of novel compound classes endowed with biological activity is at the heart of chemical biology and medicinal chemistry research. This enables novel biological insights and inspires new approaches to the treatment of diseases. Cancer cells frequently exhibit altered glycolysis and glucose metabolism and an increased glucose demand. Thus, targeting glucose uptake and metabolism may open up novel opportunities for the discovery of compounds that differentiate between normal and malignant cells. This review discusses the different chemical approaches to the development of novel inhibitors of glucose uptake through facilitative glucose transporters (GLUTs), and focusses on the most advanced and potent inhibitor classes known to date. GLUT inhibitors may find application not only in the treatment of cancer, but also of other proliferative diseases that exhibit glucose addiction.

## Graphical abstract

Potent glucose transporter (GLUT) inhibitors have been developed over the last years. This Minireview serves as an overview of the origin (natural products, natural product‐inspired, non‐natural compounds, and virtual screening) of these molecules.

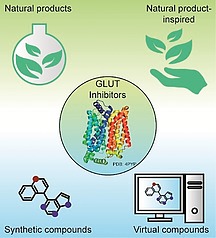

## 1. Introduction

Bioactive small molecules are the cornerstones of medicinal chemistry and chemical biology research and continue to define a large fraction of most recently approved drugs, e.g. the PI3K inhibitor Piqray (Alpelisib) for the combinatorial treatment of breast cancer.^1^ Among all drugs, natural products (NPs) hold a strong position, both historically and currently. NPs define the naturally occurring, biologically relevant chemical space and have inspired numerous concepts for the identification of bioactive compounds, including Biology‐oriented synthesis (BIOS)^2^ or the design and synthesis of pseudo‐natural products (for a discussion of the different NP‐based approaches see ref^3^).

There is a continuous demand for the identification of new biological targets for the treatment of diseases. This holds true in particular for the treatment of cancer, since cancer cells often rapidly adapt and resist to given drugs. In this respect, very recently the discovery of new compound classes that target mechanisms of tumor metabolism has spurred new research programs. This approach has had remarkable success with the clinical approval of the drug Idhifa (Enasidenib, Agios Pharmaceuticals/Celgene) in 2017, which targets a mutated form of isocitrate dehydrogenase (IDH) 2 for the treatment of relapsed/refractory acute myeloid leukemia (AML).^4^

**Figure 1:**
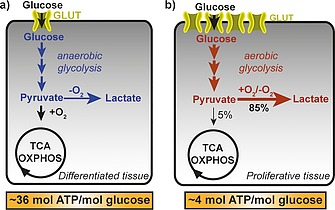
The Warburg effect. The preferred pathway for glucose metabolism in differentiated cells (a) and proliferative tissue (b). GLUT=facilitative glucose transporters; TCA=tricarboxylic acid cycle; OXPHOS=oxidative phosphorylation. Adapted from Reckzeh et al.^8^

Differentiated somatic cells perform cellular respiration, an oxygen‐dependent mitochondrial metabolic pathway, and generate thereby about 36 mol ATP per mole consumed glucose (Figure 1a).^5^ The adapted metabolism of cancer cells was first described by Otto Warburg in 1924.^6^ Warburg observed that cancer cells perform primarily glycolysis and fermentation of pyruvate to lactate in the presence of oxygen, which generates about 9‐fold less mol ATP per mol glucose (Figure 1b). Since glycolysis fuels biosynthetic pathways and thereby drives cell proliferation, interfering with glycolysis offers a promising strategy to restrict the energy and substrate supply for biosynthesis and hence cell proliferation.^7^ Addressing the first rate‐limiting step, i.e. glucose uptake, may, therefore, be a viable opportunity to initiate new medicinal chemistry programs aimed at the treatment of cancer and other diseases, which are characterized by hyperproliferation (for more details see Reckzeh et al.^8^). For glucose uptake, both normal cells and cancer cells employ facilitative transmembrane transporters termed GLUTs. Here we summarize the discovery of the currently most potent and advanced (IC_50_ < 1 µm) GLUT inhibitors (for an overview of more GLUT inhibitors regardless of their potency please refer to Granchi et al.^9^). Both NP‐derived and NP‐inspired as well as non‐NP based inhibitors are discussed.

## 2. Discovery of Novel GLUT Inhibitors

**Figure 2:**
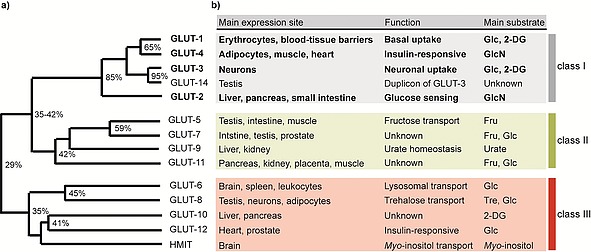
Phylogenetic GLUT similarity and functional role. a) Amino acid sequence similarities between GLUT isoforms 1–14 as obtained by CLUSTAL multiple alignment. Adapted from Scheepers et al.^10^ b) Main expression side, function and preferred substrate of GLUT class I–III.^11^ Glc = glucose, 2‐DG = 2‐deoxy‐*D*‐glucose, GlcN = glucosamine, Fru = fructose, Tre = trehalose, HMIT = Proton myo‐inositol cotransporter.

Facilitative glucose transporters (GLUTs) are transmembrane proteins that passively transport glucose or other substrates following the prevalent concentration gradient across the cell membrane. All GLUTs have a distinct expression pattern within the human organism and fulfil important and distinct functions regarding glucose homeostasis (Figure 2b). The GLUTs are subdivided into three different classes, according to their amino acid similarity (Figure 2a). Class I GLUTs 1–4 have been investigated most intensively.^10^ The different isoforms are upregulated in cancer and other diseases. Especially GLUT‐1, but also GLUT‐3, are overexpressed in most malignant tissues. To address the glucose dependence of cancer, the application of GLUT inhibitors has been proposed as a promising approach.

**Figure 3:**
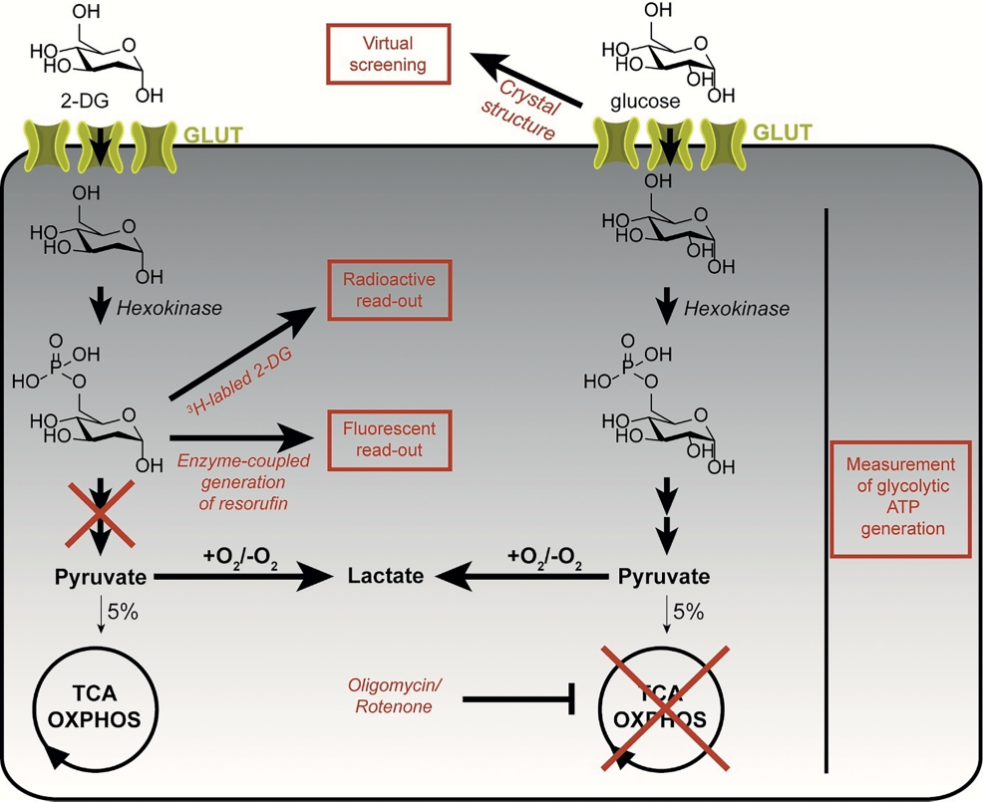
Overview of different assay principles to identify GLUT inhibitors. Glucose or 2‐DG is taken up by the cell via GLUTs and directly quantified using isotope labeling or indirectly quantified by means of enzyme‐coupled reduction of resazurin to resorufin or by quantification of the glycolytic ATP production via simultaneous treatment with inhibitors of oxidative phosphorylation (e.g. rotenone or oligomycin). Virtual screening may be performed using the crystal structure or homology models of GLUTs. GLUT = facilitative glucose transporters; TCA = tricarboxylic acid cycle; OXPHOS=oxidative phosphorylation.

**Figure 4:**
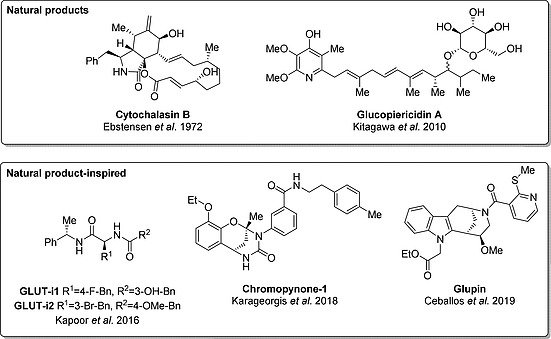
Natural products with GLUT‐inhibiting activity and natural product‐inspired GLUT inhibitors.

**Figure 5:**
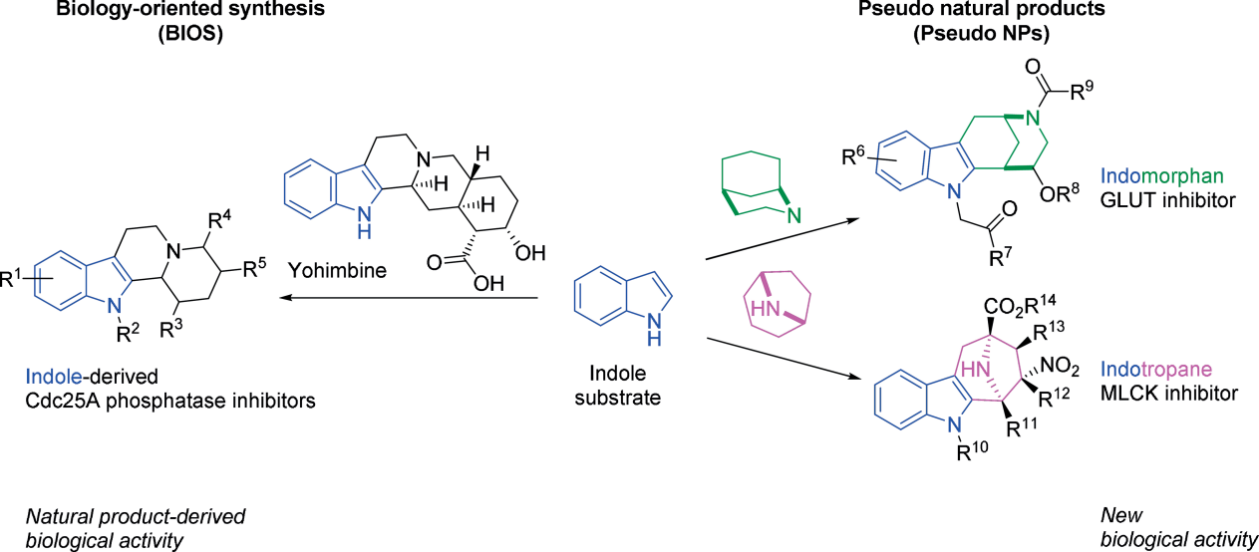
Examples for BIOS^32^ and pseudo natural product design^17^, [33c] starting from an indole.

To identify compounds that directly inhibit a protein of interest, assay systems that monitor the protein's activity in a minimal setup (e.g. only the expressed protein) are the method of choice.^12^ However, glucose transporters are transmembrane proteins that possess 12 transmembrane helices, which makes their expression and purification an unattractive endeavor. Therefore, most inhibitors of glucose transporters have been identified in cell‐based screens either in a direct fashion or indirect process (Figure 3). For direct monitoring of glucose uptake *in cellulo* 2‐deoxy‐*d*‐glucose (2‐DG) is employed, which can only be phosphorylated intracellularly by hexokinase in the 6‐position. The corresponding 6‐phosphate cannot be metabolized further and is therefore trapped inside the cell (Figure 3). For quantification, 2‐DG is radioactively labeled using tritium or ^14^C (Table 1). This method was applied to identify the inhibitory potential of cytochalasin B,^13^ glucopiercidin A,^14^ WZB117^15^ and compound 155^16^ (Figure 4, Figure 5, Table 1).

**Table 1:** Overview of a selection of highly potent glucose uptake inhibitors

Name	Entity	Compound class	Assay (Cell line)	IC_50_ (nM)
Cytochalasin B^13^	Natural product	Alkaloid, fungal metabolite	^14^C‐2DG uptake (erythrocytes)	520
Glucopiericidin A^14^	Natural product	Glucose analog	^3^H‐2DG uptake, (A431)	22
WZB117^15^	Small molecule	Polyphenol	^3^H‐2DG uptake, (A459)	500
Compound 155^16^	Small molecule	Imidazopyridine	^3^H‐2DG uptake, (HEK293,, hGLUT1^a^)	~30
GLUT‐i1^22^, GLUT‐i2^22^	Peptide analog	Phenyl amide	ATP depletion^b^,	267,
			(CHO‐K1, hGLUT‐1^a^, luciferase^c^)	140
Compound 3[20a]	Small molecule	Pyrazolopyrimidine	ATP depletion^b^, ^d^, (DLD‐1)	25
BAY‐876[21a]	Small molecule	Quinoline	ATP depletion^b^, ^d^, (DLD‐1)	2
Example 31^23^	Small molecule	Dihydrofuropyrimidine	ATP depletion^d^, ^e^, (HT1080)	10‐100
PUG‐1^25^	Small molecule	Hypoxanthine	^3^H‐2DG uptake, (CHO, hGLUT‐1^b^)	450
Chromopynone‐1^17^	Pseudo‐NP	Chromane‐tetrahydropyriminones	2‐DG uptake^f^, (HCT116)	412
Glutor^19^	Small molecule	Piperazin‐2‐one	2‐DG uptake^f^, (HCT116)	11
Glupin^18^	Pseudo‐NP	Indomorphan	2‐DG uptake^f^, (MDA‐MB‐231)	4
NV‐5440^38^	Small molecule	Bis‐piperazin‐1‐ylmethyl‐benzene	^3^H‐2DG uptake, (MCF7)	36

a Stable transfection.

b Rotenone.

c Constitutive expression.

d CellTiter‐Glo®.

e Oligomycin.

f Resazurin‐coupled.

The amount of intracellular 2‐DG that was transported via GLUTs can also be monitored indirectly, e.g. by coupling to an enzymatic reaction that leads to the generation of a fluorescence signal, e.g. resorufin generation (Table 1, Figure 3, identification of chromopynone‐1,^17^ glupin^18^ and glutor^19^). The resazurin‐resorufin detection system acts via two supplemented enzymes. The first enzyme glucose‐6‐phosphate‐dehydrogenase oxidizes 2‐DG‐6‐phosphate to 6‐phospho‐2‐deoxyglucoronic acid and thereby generates NADPH+H^+^. The second enzyme diaphorase uses the generated NADPH+H^+^ to reduce the dye resazurin to the fluorophore resorufin. Spectrometric analysis of the emission intensity of resorufin is used to estimate the amount of consumed 2‐DG.

Cellular glucose supply has also been quantified indirectly by monitoring the glycolytic ATP generation using CellTiter Glo® or transfected luciferase (Table 1, Figure 3, identification of compound 3,^20^ BAY‐876,^21^ GLUT‐i1/‐i2^22^ and example 31^23^). For this purpose, the simultaneous treatment of the cells with rotenone or oligomycin, which are known inhibitors of oxidative phosphorylation, limits the cellular ATP generation to the glycolytic pathway.

Virtual screening offers an additional possibility for *in silico* discovery of GLUT inhibitors. The crystal structures of hGLUT‐1 (inward‐open conformation) and hGLUT‐3 (outward‐open conformation) were solved by Deng et al.^24^ Homology models of hGLUT‐1–4 have been described and were used for virtual screening of GLUT inhibitors (Table 1, Figure 3, identification of PUG‐1^25^).^25^, ^26^

## 3. Natural Product GLUT Inhibitors

Cytochalasin B (Figure 4) is a fungal metabolite that is widely known for inhibition of actin polymerization.^27^ In 1972, Ebstensen et al. observed that cytochalasin B inhibits the uptake of ^14^C‐2‐DG and its incorporation in lactate with an IC_50_ value <4 µm.^13^ Moreover, the uptake of ^14^C‐2‐DG into erythrocytes is inhibited with an IC_50_ value of 0.52 µm (Table 1).^28^ Since then, this natural product often served as a positive control for glucose uptake inhibition. However, cytochalasin B was not developed further due to its primary inhibitory activity on actin polymerization.

A large class of natural products that reduce the uptake of glucose is the class of flavonoids. Phloretin was the first flavonoid that was observed to bind GLUT‐1 in a competitive manner in 1957.^29^ Since then, 15 structurally distinct flavonoids were described to bind to GLUT or inhibit glucose consumption (see e.g. Granchi et al.^9^). However, all flavonoid analogs exhibit low potency in the micromolar range, which limits their applicability as tool compounds. Other natural products that were described to block glucose uptake, also with micromolar potency, are gossypol, curcumin, resveratrol, different methylxanthines, (+)‐cryptocaryone, different ellagic acid derivatives and melatonin (see e.g. Granchi et al.^9^). The only natural product that inhibits 2‐DG uptake in the nanomolar range is glucopiericidin A (IC_50_ = 22 nm, Figure 4, Table 1).^14^ Glucopiericidin A was isolated from the broth of *Lechevalieria sp*. strain 1869–19 which had been found to inhibit filopodia protrusion. The strong inhibition of glucose uptake includes most probably the glucose moiety within glucopiericidin A, since the deglycosylated analog piericidin A is a known inhibitor of mitochondrial complex I.^30^ Glucopiercidin A inhibits the function of GLUT‐1 and GLUT‐4.^14^

## 4. Natural Product‐Inspired GLUT Inhibitors

Natural products are pre‐validated by nature for their biological activity and compound classes inspired by natural products may share this relevance. One concept to generate such compounds is biology‐oriented synthesis (BIOS). In BIOS, the complexity of a natural product is reduced in order to render it synthetically more accessible but keeping the kind of bioactivity of the guiding NP (Figure 5).^2^, ^31^ The principle has been applied successfully multiple times for the discovery of new bioactive chemical matter as illustrated with the example of yohimbine (Figure 5). However, GLUT inhibitors have not been derived from this concept.^32^

The BIOS concept has been expanded to cover larger areas of chemical space by combining it with fragment‐based compound design, to arrive at the design and synthesis of pseudo‐natural products (Figure 5).^3^, ^17^, ^18^, ^33^ In this approach NPs are reduced to fragments which still represent the properties of the guiding NPs, and fragments of different natural products then are combined de novo to yield novel combinations not occurring in nature. In contrast to BIOS, this approach is not meant to keep the original biological activity of the natural product,^34^ but to explore novel biologically relevant chemical space by combining different pre‐validated scaffolds. Hence, the combination of an indole fragment with a morphan leads to a scaffold with GLUT inhibitory properties (glupin, Figure 4, Figure 5), whereas the combination with a tropane fragment generates a compound scaffold active against myosin light chain kinase MLCK (Figure 5).^18^, [33c] Additionally, the application of this concept recently led to the identification of the novel GLUT inhibitor chemotype chroman‐tetrahydropyriminone (chromopynone‐1, Figure 4).^17^, ^18^

**Scheme 1:**
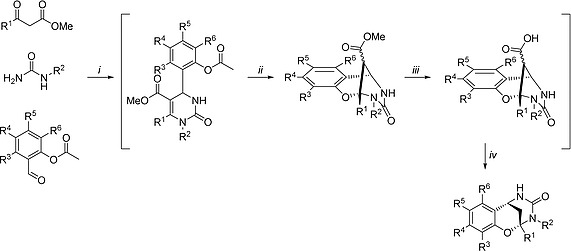
Synthesis of chromopynone‐1 and derivatives. (*i*) urea (1 equiv.), aldehyde (1 equiv.), 1,3‐dicarbonyl compound (1.5 equiv.), TMSCl (6 equiv.), DMF, r.t., 18 h; (*ii*) NaHCO_3_, MeOH/H_2_O, 40 °C, 16 h; (*iii*) LiOH (15 equiv.), THF/H_2_O, 40 °C, 18 h; (*iv*) 1 m HCl, pH 1–2, 80 °C, 6 h. Synthetic scheme adapted from Karageorgis et al.^17^

The chroman‐tetrahydropyriminone‐based chromopynone‐1 potently inhibits the uptake of 2‐DG in the colorectal cancer cell line HCT116 with an IC_50_ of 414 nm as determined via a resazurin‐coupled detection system (Figure 3, Figure 4, Table 1).^17^ It selectively targets the GLUT isoforms GLUT‐1 and GLUT‐3, since overexpression of GLUT‐1 and GLUT‐3, but not GLUT‐2 and GLUT‐4, lead to a partial rescue of 2‐DG uptake inhibition in transiently transfected CHO cells (Chinese hamster ovary).^17^ The proliferation of HCT116 cells and MIA PaCa‐2 cells (pancreatic carcinoma) was reduced with a GI_50_ value of >25 µm and 2.8 µm in the presence of 25 mm glucose, respectively. At physiological glucose concentrations (5 mm glucose), the GI_50_ values were lowered to 3.8 µm (HCT116) and 0.6 µm (MIA PaCa‐2).^17^ The synthesis of chromopynone‐1 was achieved employing a modified Biginelli multicomponent reaction, which was coupled to a stepwise intramolecular nucleophilic attack, ester hydrolysis, and decarboxylation, requiring only one final purification step (Scheme 1).^17^

The second pseudo natural product class that blocks glucose uptake by cancer cells is the class of indomorphans with their most potent member glupin (Figure 4). Glupin blocks the uptake of 2‐DG in the breast adenocarcinoma cell line MDA‐MB‐231 cells with an IC_50_ value of 4 nm (Table 1) via interaction with GLUT‐1 and GLUT‐3 (but not GLUT‐2 and GLUT‐4).^18^ Selectivity was determined in transiently overexpressing CHO cells. The inhibition of glucose uptake led to reduced glycolytic flux in MDA‐MB‐231 cells and furthermore a reduction of glycolytic metabolites in MOLT16 cells (T cell leukemia) as determined by means of metabolomic measurements.^18^ The growth of non‐malignant peripheral blood mononucleated cells and the embryonic kidney cell line IMR‐90 was not impeded after 72 h of glupin treatment (30 µm glupin, sulforhodamine B assay).^18^ However, proliferation of a variety of different cancer cell lines was reduced with IC_50_ values in the nanomolar to micromolar range.^18^

The peptide analogs GLUT‐i1 and GLUT‐i2 (Figure 4) are potent GLUT‐1/‐4 (and not GLUT‐2/‐3) inhibitors that were discovered by Kapoor et al. in a cellular ATP depletion assay using CHO‐K1 cells that were transfected with hGLUT‐1 and luciferase and simultaneously treated with the mitochondrial complex I inhibitor rotenone (Figure 3).^22^ GLUT‐i1 and GLUT‐i2 deplete the glycolytic generation of ATP with an IC_50_ value of 267 nm and 140 nm respectively (Table 1).^22^ GLUT isoform selectivity was determined by employing the colorectal adenocarcinoma cell line DLD‐1 cells (express mainly GLUT‐1), DLD‐1 *GLUT1*(–/–) cells (express mainly GLUT‐3) and CHO cells that were stably transfected with hGLUT‐2 or hGLUT‐4.^22^ The effect of this compound class on (cancer) cell growth was not determined. However, co‐crystal structures revealed that GLUT‐i1 competes with glucose at the same binding site in human GLUT‐1.^22^

## 5. Non‐Natural GLUT Inhibitors

**Figure 6:**
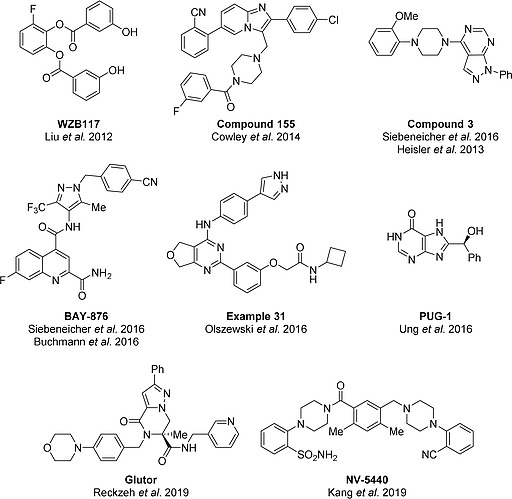
Structures of selected non‐natural GLUT inhibitors.

WZB117 (Figure 6) was identified from a compound class with antidiabetic properties.^15^, ^35^ Its inhibitory activity towards ^3^H‐2‐DG uptake was discovered using the lung carcinoma cell line A549 (IC_50_ = 500 nm, Table 1), and led to the reduction of extracellular lactate and the cellular ATP levels after 6 h to 24 h of treatment.^15^ WZB117 acts via GLUT‐1, –3 and –4 as determined in GLUT‐1/‐3/‐4‐overexpressing HEK293 cells (embryonic kidney cell line), which were used to monitor the uptake of ^3^H‐2‐DG.^36^ Competitive inhibition of GLUT‐4 as the main target was proposed as mode of action.^36^ Furthermore, treatment with WZB117 interfered with the growth and the viability of A549 cells with IC_50_ values between 10 and 30 µm, while the non‐malignant cell line NL20 (lung epithelium) was resistant.^15^ The employment of xenograft models (A549) revealed a reduction of the tumor volume by 70 % after 10 weeks of daily intraperitoneal injection (10 mg/kg) when compared to untreated control mice.^15^

In 2014, IOmet Pharma published the imidazopyridine‐based GLUT inhibitor class in a patent.^16^ The patent covers a collection of 385 compounds among which 59 derivatives were tested for their GLUT‐1–4 isoform selectivity. Most derivatives were unselective.^16^ Selectivity was determined in cHEK293 cells transiently overexpressing hGLUT‐1–4. Compound 155 (Figure 6) inhibits ^3^H‐2‐DG uptake in hGLUT‐1 transfected cHEK291 cells with an IC_50_ value of 30 nm (Table 1) and reduced the extracellular lactate level with an IC_50_ value of 90 nm in the presence of 5 mm glucose and of 400 nm in the presence of 17 mm glucose.^16^ Live‐cell imaging experiments were employed to determine the effect of treatment with compound 155 on proliferation employing a nuclear stain. After 24 h the A459 cell count was reduced with an IC_50_ value <126 nm at physiological glucose level (5 mm glucose).^16^ The effect on proliferation was weaker in the presence of 17 mm glucose (IC_50_ = 380 nm).^16^

Bayer published two compound classes in 2016 that lower the glucose uptake of cancer cells. Both were discovered in a cell‐based screen which monitors the glycolytic ATP depletion by means of a CellTiter‐Glo® assay in rotenone‐treated DLD‐1 cells (Figure 3). One inhibitor class is based on the 1*H*‐pyrazolo[3,4‐d]pyrimidine core scaffold.^20^ Compound 3 (Figure 6) induces glycolytic ATP depletion with an IC_50_ value of 25 nm (patent: example 1, Table 1).[20a] Compound 3 was selective for GLUT‐1/‐3 over GLUT‐2.[20a] Compound 3 was further characterized for its *in vitro* and *in vivo* pharmacokinetics.[20a]

The second compound class is based on the *N*‐(1*H*‐pyrazolo‐4‐yl)quinoline‐4‐carboxamide scaffold.^21^ The most active derivative BAY‐876 (Figure 6) reduces the glycolytic ATP production with an IC_50_ value of 2 nm (Table 1)[21a] and was the first compound with a strong GLUT isoform selectivity that targets GLUT‐1 but not GLUT‐2–4.[21a] BAY‐876 is a competitive inhibitor and possesses good *in vitro* and *in vivo* pharmacokinetics.[21a] Different reports about the potency of BAY‐876 to inhibit cell growth have appeared. Whereas Wu et al. could not observe an effect on the growth of different triple‐negative breast cancer cells (BT549, MDA‐MB‐436, HCC70) at 3 µm BAY‐876, Ma et al. reported inhibition of ovarian cancer growth with an IC_50_ value up to 60 nm in OVCA*R*3 cells.^37^ However, also the ovarian cancer cell line A2780 was resistant towards the treatment with BAY‐876.[37b] This indicates a cell line‐dependent sensitivity towards treatment with the GLUT‐1‐selective inhibitor. In SKOV‐3 (ovarian adenocarcinoma) xenograft mice, daily administration of 4.5 mg/kg BAY‐876 reduced the tumor volume by 68 % after two weeks.[37b]

Kadmon published a patent describing dihydrofuropyrimidine‐based GLUT inhibitors in 2015.^23^ The glycolytic ATP production of the oligomycin‐treated fibrosarcoma cell line HT1080 was inhibited by one compound (example 31, Figure 6) with an IC_50_ value between 10 and 100 nm (Table 1).^23^ Moreover, example 31 interfered with the growth of different cell lines (Jurkat (T cell lymphocytes), MOLT‐4 (T cell lymphoblast), U937 (histiocytic lymphoma)) with IC_50_ values between 256 nm and 385 nm as determined after 72 h of treatment employing a sulforhodamine B assay.^23^

**Scheme 2:**
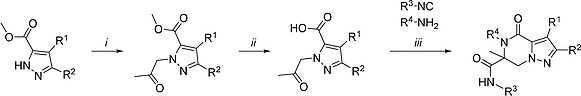
Synthetic procedure to obtain Glutor and derivatives. *i*) 1‐Chloroacetone, K_2_CO_3_, 18‐crown‐6, 1,4‐dioxane, reflux, 12–72 h; *ii*) 1–2 % NaOH, H_2_O, 70 °C, 1 h; *iii*) MeOH, r.t.‐40 °C, 5–24 h.

Waldmann et al. discovered a piperazin‐2‐one‐based compound class employing a cell‐based assay, which monitors the uptake of 2‐DG in HCT116 cells employing a resazurin‐coupled detection system (Figure 3).^19^ Glutor (Figure 6), the most active compound, inhibits the uptake of 2‐DG with an IC_50_ value of 11 nm in a GLUT‐1/‐2/‐3‐selective manner (not GLUT‐4, Table 1).^19^ Selectivity was determined by employing transiently transfected CHO cells. Treatment of different cancer cells (HCT116, UM‐UC‐3 (urinary bladder carcinoma) and BxPC‐3 (pancreatic adenocarcinoma)) with 250 nm glutor reduced the glycolytic flux after 30 min of treatment.^19^ Furthermore, glutor interfered with growth of a multitude of different cancer cell lines, most of them in the nanomolar range.^19^ UM‐UC‐3 (IC_50_ = 4 nm) and MIA PaCa‐2 cells (IC_50_ = 4 nm) were most sensitive towards the treatment.^19^ Cell growth was monitored by means of a sulforhodamine B assay after 72 h of treatment. Non‐malignant peripheral blood mononucleated cells and IMR‐90 cells were not impeded in their growth behavior.^19^ Furthermore, glutor displayed increased toxicity in the center of HCT116‐derived spheroids, a physiologically more relevant cell culture model, with an EC_50_ value of 82 nm, whereas the EC_50_ value in monolayer cultured HCT116 cells was about two times higher (EC_50_ = 193 nm).^19^ The synthesis of glutor and derivatives employs a modified Ugi four‐component reaction (*iii*, Scheme 2) in which the bifunctional core scaffold (ketone, carboxylic ester) is generated in the first two reaction steps (*i*‐*ii*, Scheme 2).

While searching for tool compounds that selectively inhibit mTORC1, Kang et al. discovered bis‐piperazin‐1‐ylmethyl‐benzene‐based GLUT inhibitors.^38^ The most active compound NV‐5440 (Figure 6) reduces mTORC1 signaling *in vitro* and *in vivo*S by blocking cellular glucose uptake with an IC_50_ of 36 nm (Table 1).^38^ The molecular target of NV‐5440 was determined via SILAC experiments to be GLUT‐1.^38^ Further investigation confirmed the inhibition of glucose uptake in a GLUT‐1–4‐dependent manner while leaving GLUT‐5 unaffected.^38^ Glycolytic metabolites were reduced upon treatment with NV‐5440 as determined by employing metabolic flux analysis.^38^ The pharmacokinetic parameters were determined *in vivo* for NV‐5440 and the metabolically more stable derivative NV‐6297.^38^

The non‐natural compound PUG‐1 was identified in an *in silico* approach employing a homology model of hGLUT‐1 (based on the *Escherichia coli* xylose transporter XylE) (Figure 6). The compound was predicted to form multiple hydrogen bond interactions within the sugar‐binding site, among others with Trp388 (transmembrane helix 10). PUG‐1 is a hypoxanthine derivative, that inhibits the uptake of ^3^H‐2DG with an IC_50_ value of 450 nm (Table 1).^25^ However, no further data describing the inhibitory potential of PUG‐1 on e.g. cancer cell growth was obtained.

A structural comparison of the highly potent GLUT inhibitors discussed here reveals that most compounds possess aromatic and non‐aromatic nitrogen‐containing heterocycles with exception of WZB117 and GLUT‐i1/‐i2 (Figure 4, Figure 6). Furthermore, the structures of the discussed GLUT inhibitors contain 2 to 5 (multi‐) cyclic structures that are linked via alkyl groups or a single bond, which offers a certain degree of flexibility (with exception of cytochalasin B). However, the binding site is only known for cytochalasin B and GLUT‐i1/‐i2 and was located at the glucose‐binding site in the inward‐open conformation of hGLUT‐1.^22^ Overlaying the electron density maps of all three inhibitors reveals that the macrolide ring of cytochalasin B overlaps with the linkers between the three aromatic rings in GLUT‐i1 and GLUT‐i2. Cytochalasin B interacts with the hydrophobic residues Trp388 (transmembrane helix 10), Trp412 (transmembrane helix 11) and Asn411 (transmembrane helix 11) and interacts with Thr137 and Trp388 via hydrogen bond interactions. The hydrophobic interaction with Trp388, Asn411, and Trp412 are also the key binding determinants for GLUT‐i1 and GLUT‐i2. Further major π–π interactions were observed between GLUT‐i1 and Trp388 as well as Phe379. Common π–π interactions between GLUT‐i1 and GLUT‐i2 that are not shared with cytochalasin B are formed with His160 (transmembrane helix 5).^22^ Direct conclusions for future inhibitor design, therefore, cannot be drawn from the selected structures of the previously developed inhibitors. However, since all described inhibitors, as well as the substrate glucose itself, interact with Trp388 in the inward‐open conformation of hGLUT‐1, this might offer a potential starting point for the *in silico* design of future GLUT inhibitors.

## 6. Summary and Outlook

This review gives an overview of the discovery of the currently most advanced and most potent GLUT inhibitors and their origin. GLUT inhibitors are gaining attention in addressing the glucose dependency in cancer and other diseases and may offer novel opportunities for future medicinal chemistry programs.

Further preclinical validation of selected GLUT inhibitors, which may be based on the compound classes described here, will validate their use as tool compounds and potential therapeutics. However, there is still an unmet need for the development of tool compounds that selectively address the more uncharacterized class II and III GLUTs in order to elucidate the function of these isoforms.

## References

[1] FDA – U. S. Food & Drug Administration, https://www.fda.gov/drugs/new-drugs-fda-cders-new-molecular-entities-and-new-therapeutic-biological-products/novel-drug-approvals-2019, **Accessed 05.09.2019**.

[2] H. van Hattum and H. Waldmann , J. Am. Chem. Soc, 2014, 136, 11853–11859. 25074019 10.1021/ja505861d

[3] G. Karageorgis , L. Laraia , D. Foley , H. Waldmann , Nat. Chem. 2019, in press. 10.1038/s41557-019-0411-x 32015480

[4] M. Galkin and B. A. Jonas , Core Evid, 2019, 14, 3–17. 31118877 10.2147/CE.S172912 PMC6503332

[5] M. G. Vander Heiden , L. C. Cantley and C. B. Thompson , Science, 2009, 324, 1029–1033. 19460998 10.1126/science.1160809 PMC2849637

[6] O. Warburg , Die Naturwissenschaften, 1924, 12, 1131–1137.

[7] M. G. Vander Heiden , Nat. Rev. Drug Discovery, 2011, 10, 671–684. 21878982 10.1038/nrd3504

[8] H. Waldmann and E. S. Reckzeh , ChemBioChem 2019, DOI: 10.1002/cbic.201900544.

[9] C. Granchi , S. Fortunato and F. Minutolo , Med. Chem. Commun, 2016, 7, 1716–1729. 10.1039/C6MD00287K PMC5198910 28042452

[10] A. Scheepers , H. G. Joost and A. Schurmann , JPEN J. Parenter. Enteral Nutr, 2016, 28, 364–371. 10.1177/0148607104028005364 15449578

[11] a) L. Szablewski , in: Glucose Homeostasis and Insulin Resistance, Bentham Science Publishers Ltd., Sharjah, U. A. E. 2012;

[12] J. Lehar , B. R. Stockwell , G. Giaever and C. Nislow , Nat. Chem. Biol, 2008, 4, 674–681. 18936752 10.1038/nchembio.120 PMC2712875

[13] R. D. Ebstensen and P. G. Plagemann , Proc. Natl. Acad. Sci. USA, 1972, 69, 1430–1434. 4338593 10.1073/pnas.69.6.1430 PMC426719

[14] M. Kitagawa , S. Ikeda , E. Tashiro , T. Soga and M. Imoto , Chem. Biol, 2010, 17, 989–998. 20851348 10.1016/j.chembiol.2010.06.017

[15] Y. Liu , Y. Cao , W. Zhang , S. Bergmeier , Y. Qian , H. Akbar , R. Colvin , J. Ding , L. Tong , S. Wu , J. Hines and X. Chen , Mol. Cancer Ther, 2012, 11, 1672–1682. 22689530 10.1158/1535-7163.MCT-12-0131

[16] P. M. Cowley , A. Wise , T. J. Brown , M. Isherwood , A. Chakrabarti (IOMET PHARMA LTD.), WO2014/187922, 2014.

[17] G. Karageorgis , E. S. Reckzeh , J. Ceballos , M. Schwalfenberg , S. Sievers , C. Ostermann , A. Pahl , S. Ziegler and H. Waldmann , Nat. Chem, 2018, 10, 1103–1111. 30202104 10.1038/s41557-018-0132-6

[18] a) J. Ceballos , M. Schwalfenberg , G. Karageorgis , E. S. Reckzeh , S. Sievers , C. Ostermann , A. Pahl , M. Sellstedt , J. Nowacki , M. A. Carnero Corrales , J. Wilke , L. Laraia , K. Tschapalda , M. Metz , D. A. Sehr , S. Brand , K. Winklhofer , P. Janning , S. Ziegler and H. Waldmann , Angew. Chem. Int. Ed. 2019, 10.1002/anie.201909518;

[19] E. S. Reckzeh , G. Karageorgis , M. Schwalfenberg , J. Ceballos , J. Nowacki , M. C. M. Stroet , A. Binici , L. Knauer , S. Brand , A. Choidas , C. Strohmann , S. Ziegler and H. Waldmann , Cell Chem. Biol. 2019, 26, 1214–1228 e1225. 31303578 10.1016/j.chembiol.2019.06.005

[20] a) H. Siebeneicher , M. Bauser , B. Buchmann , I. Heisler , T. Müller , R. Neuhaus , H. Rehwinkel , J. Telser and L. Zorn , Bioorg. Med. Chem. Lett, 2016, 26, 1732–1737; 26949183 10.1016/j.bmcl.2016.02.050

[21] a) H. Siebeneicher , A. Cleve , H. Rehwinkel , R. Neuhaus , I. Heisler , T. Müller , M. Bauser and B. Buchmann , ChemMedChem, 2016, 11, 2261–2271; 27552707 10.1002/cmdc.201600276 PMC5095872

[22] K. Kapoor , J. S. Finer‐Moore , B. P. Pedersen , L. Caboni , A. Waight , R. C. Hillig , P. Bringmann , I. Heisler , T. Müller , H. Siebeneicher and R. M. Stroud , Proc. Natl. Acad. Sci. USA, 2016, 113, 4711–4716. 27078104 10.1073/pnas.1603735113 PMC4855560

[23] K. Olszewski , J.‐I. Kim , M. Poyurovsky , K. Liu , A. Barsotti , K. Morris (Kadmon Holdings, Inc.), WO2016/210330 A1, 2016.

[24] a) D. Deng , P. Sun , C. Yan , M. Ke , X. Jiang , L. Xiong , W. Ren , K. Hirata , M. Yamamoto , S. Fan and N. Yan , Nature, 2015, 526, 391–396; 26176916 10.1038/nature14655

[25] P. M. Ung , W. Song , L. Cheng , X. Zhao , H. Hu , L. Chen and A. Schlessinger , ACS Chem. Biol, 2016, 11, 1908–1916. 27128978 10.1021/acschembio.6b00304 PMC5356226

[26] A. M. G. Thompson , O. Ursu , P. Babkin , C. V. Iancu , A. Whang , T. I. Oprea and J. Y. Choe , Sci. Rep, 2016, 6, 24240. 27074918 10.1038/srep24240 PMC4831007

[27] S. MacLean‐Fletcher and T. D. Pollard , Cell, 1980, 20, 329–341. 6893016 10.1016/0092-8674(80)90619-4

[28] T. Kasahara and M. Kasahara , Biochem. J. 1996, 315 *(Pt 1)*, 177–182. 8670104 10.1042/bj3150177 PMC1217168

[29] a) T. Rosenberg and W. Wilbrandt , Helv. Physiol. Pharmacol. Acta, 1957, 15, 168–176; 13428209

[30] a) C. Hall , M. Wu , F. L. Crane , H. Takahashi , S. Tamura and K. Folkers , Biochem. Biophys. Res. Commun, 1966, 25, 373–377; 4290528 10.1016/0006-291x(66)90214-2

[31] S. Basu , B. Ellinger , S. Rizzo , C. Deraeve , M. Schurmann , H. Preut , H. D. Arndt and H. Waldmann , Proc. Natl. Acad. Sci. USA, 2011, 108, 6805–6810. 21415367 10.1073/pnas.1015269108 PMC3084053

[32] A. Nören‐Müller , I. Reis‐Corrêa Jr , H. Prinz , C. Rosenbaum , K. Saxena , H. J. Schwalbe , D. Vestweber , G. Cagna , S. Schunk , O. Schwarz , H. Schiewe and H. Waldmann , Proc. Natl. Acad. Sci. USA, 2006, 103, 10606–10611. 16809424 10.1073/pnas.0601490103 PMC1502279

[33] a) A. Christoforow , J. Wilke , A. Binici , A. Pahl , C. Ostermann , S. Sievers and H. Waldmann , Angew. Chem. Int. Ed. 2019, 10.1002/anie.201907853; PMC7687248 31339620

[34] B. Over , S. Wetzel , C. Grutter , Y. Nakai , S. Renner , D. Rauh and H. Waldmann , Nat. Chem, 2013, 5, 21–28. 23247173 10.1038/nchem.1506

[35] W. Zhang , Y. Liu , X. Chen and S. C. Bergmeier , Bioorg. Med. Chem. Lett, 2010, 20, 2191–2194. 20194024 10.1016/j.bmcl.2010.02.027

[36] O. A. Ojelabi , K. P. Lloyd , A. H. Simon , J. K. De Zutter and A. Carruthers , J. Biol. Chem, 2016, 291, 26762–26772. 27836974 10.1074/jbc.M116.759175 PMC5207184

[37] a) Q. Wu , D. Heidenreich , S. Zhou , S. Ackloo , A. Krämer , K. Nakka , E. Lima‐Fernandes , G. Deblois , S. Duan , R. N. Vellanki , F. Li , M. Vedadi , J. Dilworth , M. Lupien , P. E. Brennan , C. H. Arrowsmith , S. Müller , O. Fedorov , P. Filippakopoulos and S. Knapp , Nat. Commun, 2019, 10, 1915; 31015424 10.1038/s41467-019-09672-2 PMC6478789

[38] S. A. Kang , D. J. O'Neill , A. W. Machl , C. J. Lumpkin , S. N. Galda , S. Sengupta , S. J. Mahoney , J. J. Howell , L. Molz , S. Hahm , G. P. Vlasuk and E. Saiah , Cell Chem. Biol. 2019, 26, 1203–1213 e1213. 31231029 10.1016/j.chembiol.2019.05.009

